# Influenza-Specific T Cells from Older People Are Enriched in the Late Effector Subset and Their Presence Inversely Correlates with Vaccine Response

**DOI:** 10.1371/journal.pone.0023698

**Published:** 2011-08-22

**Authors:** Lisa E. Wagar, Beth Gentleman, Hanspeter Pircher, Janet E. McElhaney, Tania H. Watts

**Affiliations:** 1 Department of Immunology, University of Toronto, Toronto, Canada; 2 Department of Medicine, University of British Columbia, Vancouver, Canada; 3 Department of Immunology, Institute of Medical Microbiology and Hygiene, Freiburg, Germany; 4 Center for Immunotherapy of Cancer and Infectious Diseases, University of Connecticut School of Medicine, Farmington, Connecticut, United States; University of Georgia, United States of America

## Abstract

T cells specific for persistent pathogens accumulate with age and express markers of immune senescence. In contrast, much less is known about the state of T cell memory for acutely infecting pathogens. Here we examined T cell responses to influenza in human peripheral blood mononuclear cells from older (>64) and younger (<40) donors using whole virus restimulation with influenza A (A/PR8/34) ex vivo. Although most donors had pre-existing influenza reactive T cells as measured by IFNγ production, older donors had smaller populations of influenza-responsive T cells than young controls and had lost a significant proportion of their CD45RA-negative functional memory population. Despite this apparent dysfunction in a proportion of the older T cells, both old and young donors' T cells from 2008 could respond to A/California/07/2009 ex vivo. For HLA-A2+ donors, MHC tetramer staining showed that a higher proportion of influenza-specific memory CD8 T cells from the 65+ group co-express the markers killer cell lectin-like receptor G1 (KLRG1) and CD57 compared to their younger counterparts. These markers have previously been associated with a late differentiation state or immune senescence. Thus, memory CD8 T cells to an acutely infecting pathogen show signs of advanced differentiation and functional deterioration with age. There was a significant negative correlation between the frequency of KLRG1^+^CD57^+^ influenza M1-specific CD8 T cells pre-vaccination and the ability to make antibodies in response to vaccination with seasonal trivalent inactivated vaccine, whereas no such trend was observed when the total CD8^+^KLRG1^+^CD57^+^ population was analyzed. These results suggest that the state of the influenza-specific memory CD8 T cells may be a predictive indicator of a vaccine responsive healthy immune system in old age.

## Introduction

The aging human immune system is characterized by a variety of functional changes. In particular, the T cell population undergoes dramatic alterations in old age. Thymic involution results in a greatly diminished capacity to produce new naïve T cells [1], [2]. Exposure to common viruses such as human cytomegalovirus (CMV) and to a lesser extent Epstein-Barr virus (EBV), which are unable to be cleared by the immune system, can result in the accumulation of oligoclonal T cell expansions [3]–[8]. The human immune system must balance control of chronic viral infection and over-activation of pathogenic inflammatory processes [9]. In chronically infected hosts, the increased competition for both space and resources in the T cell compartment leads to fewer CD8 T cells which are able to respond to other antigens. The combined decrease in naïve T cell output and repertoire as well as enhanced expansion of select memory/effector T cell clones can greatly reduce the ability to mount responses against new pathogens. According to a longitudinal study of over one hundred older people, approximately 9/10 elderly individuals are estimated to be CMV-infected; the immune risk phenotype associated with CMV expansions is a predictor of mortality in older individuals [4]. Although some of the above alterations have been used to predict poor outcome, we still know relatively little about what constitutes a “healthy” immune system as we age [10].

Chronically stimulated T cells lose their functional capacity over time, with upregulation of inhibitory receptors and decreased cytolytic function [9], [11], [12]. Expression of killer cell lectin-like receptor G1 (KLRG1), a proposed marker of proliferative senescence, is increased on both mouse and human T cells following chronic antigenic stimulation as well as on CMV-specific CD8 T cell clones [13]–[15]. Moreover, co-expression of KLRG1 with another inhibitory receptor, CD57, more clearly defines the population of CD8 T cells which are capable of producing cytokines but unable to proliferate upon activation [16]. On the other hand, CD8 T cells from healthy adults that are specific for an acutely infecting pathogen, influenza, do not express significant levels of KLRG1 [15]. Although memory T cells from chronic infections have been extensively studied in humans, little is known about the effect of age on the phenotype of memory T cells against an acutely infecting pathogen and how this impacts on their ability to mount a protective response.

Influenza represents a significant disease burden in the elderly population. Individuals 65 and over account for the majority (almost 90%) of influenza and influenza-related pneumonia deaths [17]. An estimated 1.4 to 16.7 deaths per 100 000 people occur annually due to influenza infections or related complications in the United States [17]. Influenza is a single-stranded RNA virus that infects lung epithelial cells; binding is mediated through influenza surface hemagglutinin (HA) protein. The seasonal influenza trivalent inactivated vaccine (TIV) primarily elicits an antibody response targeted against the globular head of HA. Although neutralizing antibodies generated against HA can protect from infection with a specific strain, these antibody responses are generally not cross-reactive with other influenza strains [18]. However, cross-strain protection can occur from the generation of memory T cells [18], [19], as T cell epitopes are often found in internal proteins, such as matrix protein 1, nucleoprotein, and the PB subunit of influenza polymerase which are less susceptible to mutation and are widely conserved between influenza strains [20]–[22]. For this reason, CD8 T cells are considered critical for the control of influenza infection, particularly in the absence of sufficient titers of neutralizing antibodies [18], [23]. Due to the seasonal nature of influenza incidence, individuals may be acutely infected multiple times over the course of their lifetime with a variety of influenza A strains, primarily of the H3N2 and H1N1 subtypes. This presents a unique opportunity to examine a repeatedly boosted T cell response to an acute viral infection in the human population.

A number of studies have attempted to identify correlates of protection to distinguish individuals who are at greater risk of acquiring a severe influenza infection. Studies have confirmed that antibody titers and T cell responses post-vaccination with TIV do not correlate with one another [24], [25]. Although some literature has suggested that the cell-mediated compartment is less functional in older donors, these studies were done on bulk peripheral blood mononuclear cells (PBMC) and did not definitively identify a T cell population responsible for producing the protective or detrimental factors [25], [26]. To address this issue, a cohort of healthy young and older donors was recruited to examine the state of their cross-reactive influenza-specific memory T cells. The results show that resting, unstimulated influenza-specific memory T cells from older donors are more differentiated than their younger counterparts. Moreover, a more functional influenza-specific CD8 T cell population and a lower frequency of KLRG1^hi^CD57^hi^ influenza M1-specific CD8 cells predict a stronger antibody response to subsequent immunization with the trivalent influenza vaccine (TIV). Interestingly, T cells from both young and older donors recruited in 2008 not only responded to influenza A/PR8 but showed similar levels of reactivity to the A/2009 H1N1 strain, suggesting that the residual functional T cells in the older population may well be capable of providing protection to influenza strains sharing the conserved T cell epitopes.

## Materials and methods

### Ethics statement

All donors gave written informed consent and ethics approval was granted by both institutions involved in the study, including the Clinical Research Ethics Board of the University of British Columbia, approved protocol H07-02008-A009 and the Research Ethics Board of the University of Toronto, approved protocol number 22752.

### Study design and sample collection

Forty-four older donors (28 donors aged 65–74, 16 donors aged 75+) and 12 young controls (aged 20–40) were recruited at the Vancouver Coastal Health Research Institute (Vancouver, B.C.) prior to the 2008–2009 influenza season. Donors were vaccinated with a standard dose of the 2008–2009 Sanofi Pasteur Vaxigrip® seasonal TIV. Peripheral blood was collected pre-vaccination and 4, 10, and 20 weeks post-vaccination. PBMC were isolated by Ficoll density gradient centrifugation and stored at −150°C prior to use. None of the donors recruited for this study had a confirmed influenza infection during the 2008/2009 season. Identification of HLA-A2 positive donors was performed by flow cytometry on PBMC with an HLA-A2-specific labeled antibody (clone BB7.2).

### Viruses and media

Stocks of influenza A/Puerto Rico/8/34 (PR8) were generated by propagation in embryonated chicken eggs. Influenza A/California/7/2009-like virus was produced using an MDCK cell line system and was kindly provided by Dr. Jonathan Gubbay at the Public Health Ontario Laboratory in Toronto. Lymphocytic choriomeningitis virus (LCMV) Armstrong strain was kindly provided by Dr. Pamela S. Ohashi, University Health Network, Toronto, and propagated in the baby hamster kidney (BHK-21) cell line (American type culture collection line CCL10). Complete PBMC medium used for this study consisted of RPMI 1640 HEPES modification (Sigma-Aldrich, St. Louis, MO) containing 10% fetal bovine serum (FBS, Invitrogen), 1% non-essential amino acids (Invitrogen), 100 U/mL penicillin, 0.1 mg/mL streptomycin, 1 mM L-glutamine, 1 mM sodium pyruvate, and 0.1% β-mercaptoethanol.

### Hemagglutination inhibition assay

Antibodies against the specific influenza strains contained in the vaccine formulation were tested by hemagglutination inhibition (HI) assay. Briefly, serum was drawn from 5 cc of whole clotted blood that had been centrifuged in an untreated vacutainer tube and was frozen until use. Influenza-specific antibodies were detected in serum by HI assay as previously described [27].

### Assessment of T cell responses to live influenza virus challenge

PBMC were resuspended in complete medium to 1×10^7^ cells/mL and plated at 0.1 mL/well in a microtiter round-bottom plate. Cell suspensions were infected with 5 hemagglutination units (HAU)/well of influenza virus or 2000 PFU/well of LCMV Armstrong strain and maintained at 37°C, 5% CO_2_ for 18 hours. Brefeldin A (BD Biosciences) was added to all wells at a final concentration of 1.5 µL/mL for intracellular cytokine accumulation during the last 6 hours of stimulation.

### Influenza-specific tetramer staining in HLA-A2 donors

PBMC were thawed in complete medium and resuspended to 4×10^6^ cells/well in a round-bottom microtiter plate. Biotinylated MHC I/peptide monomers (obtained from the University of Montreal immunophenotyping laboratory) were conjugated with extravidin-PE (Sigma-Aldrich) to create tetramers. The tetramer used for HLA-A2 donors was M1 (GILGFVFTL). Cells were stained with surface markers and tetramer concurrently for one hour at 4°C in PBS with 3% FCS, 1% sodium azide.

### Flow cytometry

The following anti-human mAbs were used: CD27, CD28, CD8, CD3, IFNγ, CD45RA, TNFα (eBioscience); CD57 (BD Biosciences); granzyme B (Invitrogen); and KLRG1 [28] (clone 13F12F2). For intracellular cytokine detection, cells were first stained for surface markers, fixed and permeabilized with Cytofix/Cytoperm (BD Biosciences), then stained for intracellular markers for 30 minutes at 4°C. Fluorescence intensity was measured using an LSR II (BD Biosciences). Approximately 4×10^6^ total PBMC were used for the viral stimulation assay per condition. The entire sample was collected during flow cytometry, resulting in a typical range of 3×10^4^ to 1.3×10^5^ CD8 T cell events collected per condition, with fewer CD8 T cells observed in samples isolated from older donors. Direct ex vivo tetramer staining produced approximately 1×10^5^ to 5×10^5^ CD8 T cell events per sample. Fluourescence minus one (FMO) controls were used to determine background levels of staining. For tetramer staining, gates were placed based on both FMO and analysis of contours from the total CD8 T cell population. If the tetramer gate was placed only on the tetramer bright, rather than the total tetramer population (relative to FMO), the phenotypic trends observed were identical, but due to the limited number of events, statistical significance was weaker. Data were analyzed using FlowJo software (Tree Star) and SPICE version 5 [29] (available from the National Institute of Allergy and Infectious Diseases).

### Statistical Analysis

When comparing only two groups, an unpaired, two-tailed t-test was performed. For comparing more than two groups, a one-way analysis of variance (ANOVA) followed by a Tukey test was used. Correlations between two variables were assessed for significance by Pearson correlation. A 95% confidence interval was selected for each test.

## Results

### The functional recall T cell response to influenza differs in frequency between the old and the young

The matrix and nucleoprotein epitopes in influenza A viruses have changed little between 1934 and the present day [21]. To avoid confounding issues from responses to recent seasonal-specific responses and to capture as much of the cross-reactive conserved T cell response as possible, we used the H1N1 strain from 1934, A/PR8, for ex vivo stimulation since it would allow us to assess recall CD8 T cell responses to influenza virus independently of HLA type over a wide range of ages in the study population. As IFNγ is the predominant cytokine produced by CD8 T cells responding to viruses, we used IFNγ positive cells to identify influenza reactive T cells. Forty-four older donors (28 donors aged 65–74, 16 donors aged 75+) and 12 young controls (aged 20–40) were recruited prior to the 2008–2009 influenza season and vaccinated with seasonal influenza vaccine as described in the methods.

**Figure 1:**
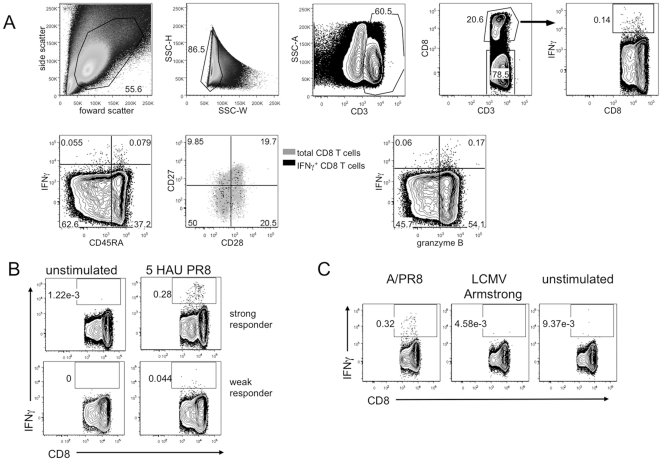
Flow cytometry gating strategy and specificity controls for detection of CD8^+^ and CD8^−^ T cells producing IFNγ in response to live influenza virus challenge. Total PBMC from under 40 years old donors and 65+ donors were stimulated with influenza A/PR8 for 18h as described in the methods, then stained for intracellular IFNγ and analyzed by flow cytometry. Influenza-responsive T cells were considered to be those that produced IFNγ after 18 hours of stimulation. Gates were drawn based on FMO controls. (A) Representative gating of flow cytometry data collected from total PBMC of an older donor stimulated with PR8. (B) CD8 T cells from two donors representing strong (*top panels*) and weak (*bottom panels*) IFNγ responses to PR8 stimulation. (C) CD8 T cell IFNγ responses to influenza virus stimulation (*left*), an irrelevant control virus, lymphocytic choriomeningitis virus Armstrong strain (*middle*) or without stimulation (*right*) for 18 hours.

In pilot studies we found that maximal IFNγ production was observed between 12 and 18 hours of stimulation with 5 HAU of PR8 (data not shown). Fig. 1A shows representative gating for a single donor stimulated with influenza virus for 18h. After gating on T cell populations based on CD3 and CD8 staining, phenotype and functional marker gates were determined based on FMO controls. T cells which produced IFNγ above the FMO background were considered to be influenza responsive. IFNγ values reported for this study are the difference of the IFNγ percentage in the PR8-stimulated culture and unstimulated total PBMC control. Responses to A/PR8 varied between donors, with responses as high as 0.93% in the CD8 T cell compartment (representative donors shown in Fig. 1B). To control for non-specific responses of the PBMC to a viral infection, we also stimulated the human PBMC with a rodent RNA virus, LCMV Armstrong, as a control, as most humans would not be expected to have been exposed to this virus. Indeed for those donors tested, responses to LCMV were similar to unstimulated control (Fig. 1C); therefore for the remainder of the study we used the unstimulated condition to measure background IFNγ production. Although the frequency and number of IFNγ^+^ events was quite small, particularly in older donors, by examining a large cohort of donors, we could identify a consistent phenotypic pattern in young and older PBMC samples.

**Figure 2:**
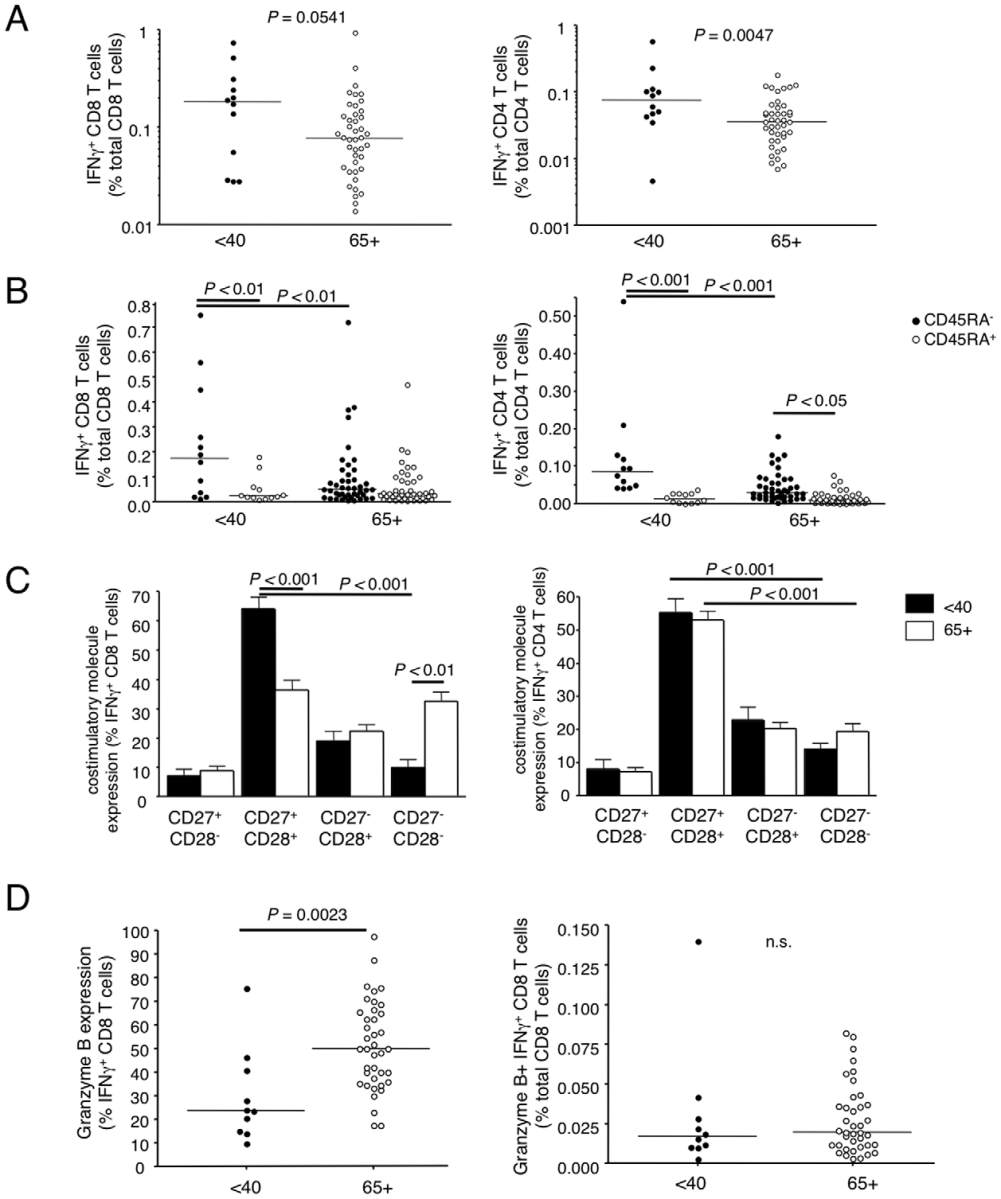
Older donors have fewer influenza-responsive T cells prior to vaccination and a higher proportion of these T cells are of a late effector phenotype than those in younger donors. PR8-stimulated T cells were phenotyped for memory and effector T cell markers CD45RA, CD28, and CD27 in young, under 40 and older, 65+ donors. CD8 T cells were also examined for granzyme B expression after stimulation (flow cytometry gating as in Figure 1). (A) Proportions of influenza functionally responsive CD8 (*left*) and CD4 (*right*) T cells out of total CD8 and CD4 pools. (B) Division of influenza responsive CD8 and CD4 T cells into CD45RA^−^ memory and CD45RA^+^ late effector cell subsets. (C) Costimulatory molecule expression profiles in young and older donors in influenza responsive CD8 and CD4 T cells. (D) Granzyme B expression within the influenza responsive IFNγ^+^ CD8 T cell subset (*left*) and overall proportions of granzyme B^+^IFNγ^+^ CD8 T cells out of the total CD8 T cell population (*right*).

Having optimized the ex vivo restimulation assay, we compared the frequency of influenza reactive T cells in PBMC from older and younger donors taken prior to vaccination. By gating on the CD8^+^ or CD8^−^ CD3 populations, we compared both CD8 and CD4 T cells (Fig. 2A). In general, donors 65 and older showed a reduced frequency of influenza reactive T cells following ex vivo stimulation with influenza virus (*P* = 0.054 for CD8 T cells and *P* = 0.005 in the CD4 populations).

### Influenza reactive T cells from the older donors are of a late effector phenotype

Memory T cell subsets can be categorized based on their expression of specific CD45 isoforms [30]–[32]. Naïve T cells express the CD45RA isoform, which is then replaced with CD45RO upon conversion to the memory population. However, late effector T cells can re-express CD45RA in the absence of the RO isoform [30]–[34].

It has been previously demonstrated that stimulation of influenza-specific T cells does not result in detectable cell division before day 4 in culture [35]. As PBMC were stimulated for a maximum of 18 hours, influenza-specific IFNγ positive cells that we detect cannot come from a naïve population. Thus CD45RA staining was used to distinguish memory T cells from late effector cells. In young donors, the majority of IFNγ^+^ CD8 and CD4 T cells are of the memory CD45RA^−^ phenotype (Fig. 2B). However, in the older group, the frequency of the CD45RA^−^ memory population of T cells is significantly reduced, resulting in an enrichment of the CD45RA^+^ population. Based on the proportion of CD45RA^+^ influenza-responsive T cells, it appears that late effector T cells could not compensate in the functional T cell repertoire for the loss of the memory subset in older donors.

Another marker of differentiation is the presence or absence of the costimulatory receptors CD28 and CD27. CD27 and CD28 are highly expressed on naïve T cells, with some expression maintained or re-activated upon stimulation in early memory T cell stages. However, their expression can be attenuated by the late stage differentiation of human CD8 T cells depending on their specificity [30]–[34], [36]–[39]. Moreover, CD27 has been shown to be critical for maintaining telomerase activity in human CD28^−^ memory CD8 T cells [40]. Most influenza reactive T cells from the young donors express both CD28 and CD27 on their surface (Fig. 2C). In contrast, CD8 T cells from the older population showed a dramatic loss of the CD28 CD27 double positive population and a gain of CD28 CD27 double negative CD8 T cells (Fig. 2C).

Granzyme B expression was also measured in the CD8 T cell populations. Granzyme B is expressed in memory CD8 T cells and is important for mediating the killing of virus-infected cells [41], [42]. We found that granzyme B was expressed in a greater proportion of the influenza reactive IFNγ positive CD8 T cells from older donors, however, as has been shown by others [26], granzyme B is also overexpressed in the total CD8 T cell population of 65+ donors. Overall, the frequency of CD8 T cells which were dual positive for granzyme B and IFNγ out of the total CD8 T cell population did not differ between young and older donors (Fig. 2D).

Taken together, the loss of influenza-specific CD45RO memory T cells and the enrichment of CD27^−^CD28^−^ CD8 influenza specific T cells suggests that the less differentiated memory population is decreased in the older population, resulting in an increased proportion of the late effector subset.

### HLA-A2 influenza M1 tetramer positive memory T cells show evidence of immune dysfunction and senescence

**Figure 3:**
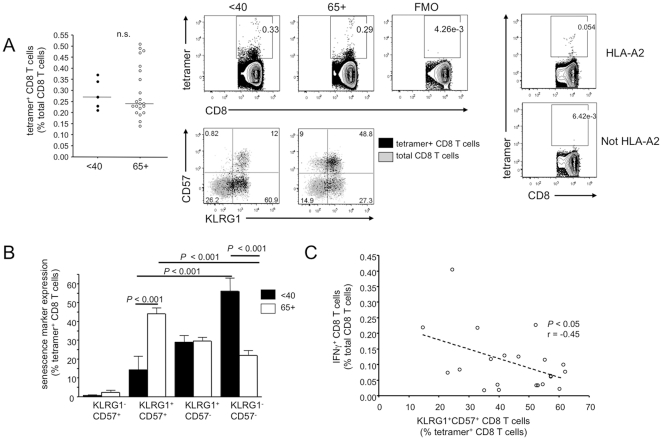
Influenza-specific T cells from older donors have a late effector phenotype and express markers associated with replicative senescence. Total unfractionated PBMC from HLA-A2 donors were stained directly ex vivo for the M1-specific T cell receptor by tetramer and for the markers KLRG1 and CD57. (A) Tetramer positive CD8 T cells from HLA-A2 positive young and older adults. Representative staining is shown for KLRG1 and CD57 in the tetramer positive subset overlaid on the total CD8 T cell population in a young and 65+ donor. The rightmost panels show A2/M1 tetramer staining on an A2 and a non-A2 donor, to confirm tetramer staining specificity. This control was repeated with 3 A2 positive and 3 A2 negative donors with similar results. (B) KLRG1 and CD57 phenotypes in the tetramer positive influenza-specific CD8 T cell population from under 40 and 65+ HLA-A2 positive donors. (C) Comparison of IFNγ expression in CD8 T cells stimulated for 18 hours with PR8 and the relative proportion of tetramer positive CD8 T cells which co-express KLRG1 and CD57 directly ex vivo on a per-donor basis in HLA-A2 positive older (65+) donors.

The finding that influenza-specific T cells in 65+ donors show signs of terminal differentiation was based on their functional ability to produce IFNγ upon restimulation. Therefore, we sought to analyze the phenotype of the influenza-specific resting memory T cell pool independently of restimulation. As about half the donors in the cohort were HLA-A2 positive, we used an HLA-A2 tetramer containing the immunodominant influenza M1_58–66_ epitope to characterize the influenza-specific memory population directly ex vivo (Fig. 3). The tetramer positive population identified based on FMO controls showed a weakly staining population as well as a tetramer bright population. Control experiments (Fig. 3A right) with HLA-A2-negative donors showed that this tetramer population is specific to the HLA-A2 positive donors. It is possible that the tetramer dim population represents T cells with a lower level of TCR. Although the proportion of tetramer positive CD8 T cells specific for influenza M1 varied between donors, in contrast to the functional results, the frequency of these CD8 T cells was largely unaffected by age (Fig. 3A left).

The cell surface proteins KLRG1 and CD57 have previously been reported to be co-expressed on replicatively senescent memory and effector T cells and are often present on T cells specific for chronic latent infections such as human cytomegalovirus [13], [14], [16]. We thus aimed to ascertain whether these senescence markers could also be co-expressed on memory CD8 T cells generated from an acute influenza infection. Indeed, almost half of all tetramer positive influenza-specific CD8 T cells from older HLA-A2 donors exhibited dual expression of KLRG1 and CD57 immediately ex vivo (Fig. 3B). This was also observed when we gated only on the tetramer bright population (data not shown). Young donors had significantly larger proportions of non-senescent KLRG1^−^CD57^−^ CD8 T cells, while KLRG1^+^CD57^+^ cells were very rare in this age group.

As co-expression of KLRG1 and CD57 are thought to mark senescent CD8 T cells, we analyzed the correlation between the proportion of the tetramer^+^ cells which were dual positive for senescence markers with the donor's ability to produce IFNγ in response to 18h PR8 virus stimulation (Fig. 3C). A negative correlation was found in older HLA-A2 donors between the proportion of KLRG1^+^CD57^+^ influenza-specific T cells and the ability to functionally respond to influenza virus in vitro. No such correlation was found in the young controls, for which the mean percentage of CD8 T cells with co-expression of senescence markers was less than 15%.

The inhibitory receptors Programmed Death 1 (PD-1) and T cell immunoglobulin domain and mucin domain 3 (Tim-3) are over-expressed in chronic infections including LCMV in mouse [43], [44] and HIV [45]–[49] and HCV [50] in humans. We therefore analyzed whether these same markers are expressed on influenza-specific CD8 T cells as a consequence of normal aging. Tetramer positive cells were negative for Tim-3 and expressed only slightly elevated levels of PD-1 that, although higher than FMO controls, were indistinguishable from background levels detected on total CD8 T cells (Fig. S1). Thus the KLRG1^+^CD57^+^ influenza- specific T cells, while resembling senescent cells specific for the persistent pathogen CMV, are distinct from the exhausted T cells observed in chronic HIV or HCV infection.

Taken together, the results show that although age does not seem to affect the proportion of influenza M1-specific CD8 T cells within the total CD8 T cell pool, these cells express markers associated with immune senescence, and this correlates with the lower frequency of IFNγ production in response to live influenza virus in the older group.

### CD4 and CD8 T cell responses to live influenza virus pre-vaccination predict the ability to generate robust antibody responses upon vaccination with TIV

**Figure 4:**
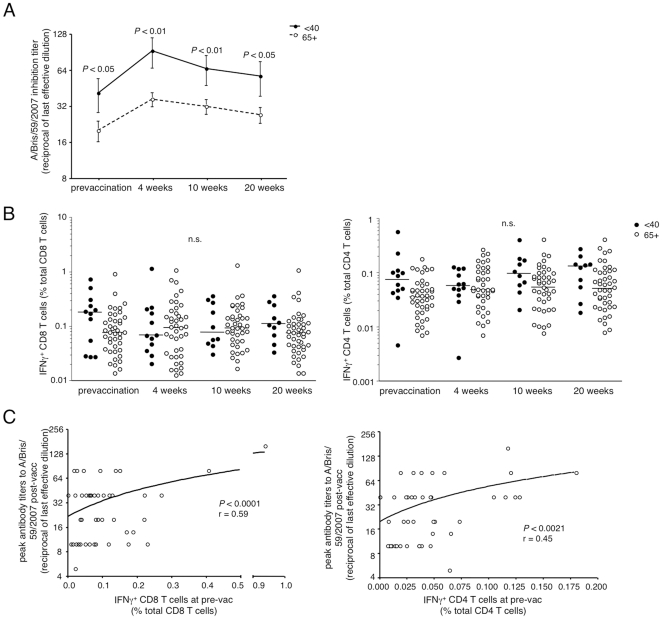
An effective T cell response to whole influenza stimulation prevaccination predicts higher peak antibody titers post-vaccination. Antibody production pre-vaccination and four, ten, and twenty weeks post-vaccination with TIV was measured by hemagglutinin inhibition assay. Titers against A/Brisbane/59/2007, an H1N1 strain contained in the seasonal vaccine, are shown. T cell responses post-vaccination were measured as described in Figure 1 for pre-vaccination samples. (A) Antibody responses in serum from young (•, solid line) and 65+ (○, dashed line) donors against A/Brisbane/59/2007 (H1N1) before and after vaccination with TIV. (B) CD8 (*left*) and CD4 (*right*) T cell responses to PR8 before and after vaccination as measured by IFNγ cytokine production. (C) Correlation between IFNγ^+^ CD8 (*left*) and CD4 (*right*) T cells present at pre-vaccination and peak antibody titers post-vaccination with TIV in older donors.

It is well documented that the ability to generate antibodies upon vaccination is diminished with age [51]–[53]. Analysis of antibody responses following immunization of this cohort confirmed this finding, with considerably lower peak antibody titers produced against the seasonal H1N1 strain, A/Brisbane/59/2007, which was contained in the vaccine (Fig. 4A). Although antibody responses were weaker in the 65+ donors overall, a heterogeneity in peak titers was observed.

To determine whether vaccination altered the cross-reactive functional T cell response, we examined the ability of PBMC to respond to PR8 virus ex vivo in the cohort post-vaccination and compared the T cell response to the pre-vaccination analysis. We observed no major changes in the proportions of functional influenza-specific CD8 and CD4 T cell responses of young or older donors post-vaccination (Fig. 4B), indicating that there is little T cell response to this vaccine.

Although the cross reactive T cell responses to influenza A/PR8 were relatively unaffected by the TIV, the analysis of the phenotype and quantity of the T cell response prior to vaccination offered the possibility of asking whether the pre-vaccination influenza-specific T cell population can predict the response to vaccination (as measured by antibody titers from the hemagglutination inhibition assay). Indeed, the frequency of functional influenza-specific CD4 and CD8 T cell responses in the older group pre-vaccination correlated with the subsequent antibody response post-vaccination (Fig. 4C), with the caveat that the correlation is lost between CD8 T cell responses and antibody responses if the two highest IFNγ responders are removed.

**Figure 5:**
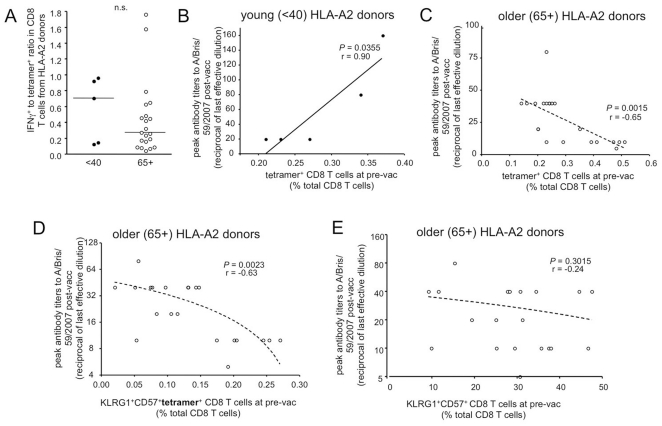
The functional influenza repertoire diminishes with age compared to the pool of influenza-specific CD8 T cells and the proportion of KLRG1^hi^ CD57^ hi^ influenza-specific T cell pre-vaccination inversely correlates with vaccine responsiveness. IFNγ positive populations were identified by influenza virus challenge as described in the methods. Influenza-specific CD8 T cell populations were identified by tetramer staining direct ex vivo for HLA-A2 donors. Comparisons between IFNγ and tetramer were made on a per-donor basis from pre-vaccination PBMC samples of young and older donors. (A) Ratio of IFNγ positive and tetramer positive CD8 T cells at pre-vaccination. (B) Comparison of peak antibody titers against seasonal H1N1 post-vaccination and total influenza-specific CD8 T cells out of total CD8 T cells present at pre-vaccination in young donors. (C) Correlation between peak H1N1 antibody titers induced from TIV vaccination and pre-existing tetramer^+^ CD8 T cells specific for influenza in older HLA-A2 donors. (D) The proportion of pre-existing CD8 T cells which were triple positive for KLRG1, CD57, and tetramer staining in 65+ donors inversely correlates with their peak H1N1 antibody titer production post-vaccination with TIV. (E) Lack of correlation between peak H1N1 antibody titers and frequency of KLRG1^+^CD57^+^ CD8 T cells in the total CD8 population.

Although there was a trend in young donors toward a proportionally more functional population compared to the older group, this finding was not statistically significant (Fig. 5A). We found however, that for the younger group, an enhanced pre-vaccination population of tetramer^+^ CD8 T cells strongly correlated with successful antibody production upon vaccination with TIV (Fig. 5B). Surprisingly, an inverse correlation was found in the 65+ group, with poor antibody responses in donors who had the largest populations of influenza-specific CD8 T cells prior to vaccination (Fig. 5C). However, upon further examination of older donors, we observed that many of the tetramer^+^ CD8 T cells were KLRG1^+^CD57^+^, and that an increased frequency of these KLRG1^+^CD57^+^ influenza-specific cells correlated with a poor vaccination outcome for H1N1 (Fig. 5D). No such correlation was found when the KLRG1^+^CD57^+^ population from the total CD8 T cell population was compared with peak antibody titers post-vaccination, arguing that it is the influenza-specific T cell memory phenotype that is predictive of vaccine outcome (Fig. 5E).

### Functionally competent memory CD8 T cells from 2008 samples cross-react with A/California 2009 influenza

We have shown that older donors are more likely to have a late effector population of influenza-responsive T cells, and that many of the CD8 T cells specific for influenza show a terminally differentiated phenotype. Nevertheless, at least 30% of the influenza M1 tetramer positive cells in the older group remained KLRG1 CD57 double negative, and should maintain the ability to respond to infection. Moreover, functional (IFNγ secreting) CD8 T cells could be identified after restimulation of PBMC from the older donors and about 35% of these were of the less differentiated CD28^+^CD27^+^ phenotype. Therefore, these cells have the potential to protect against subsequent infection.

**Figure 6:**
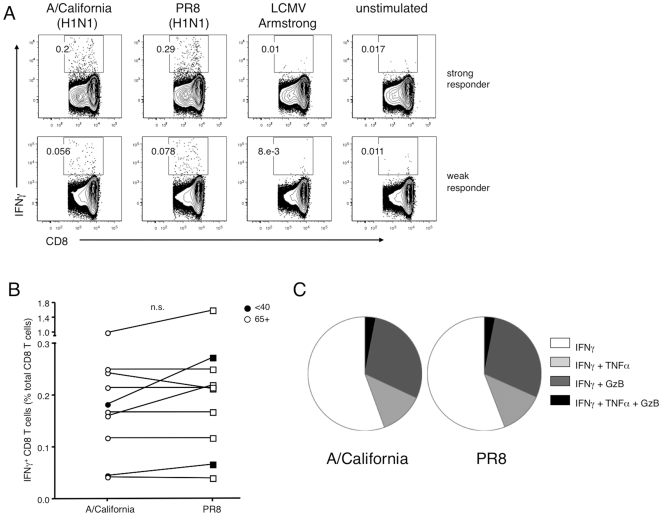
Older donors can mount a T cell response against novel influenza strains despite expression of a significant fraction of influenza-specific T cells that express markers of late differentiation and senescence. Ten donors (2 under 40, 8 donors 65+) from the cohort were selected for in vitro stimulation with A/California/7/2009-like influenza virus. Donor PBMC from either the pre-vaccination or 4 week post-vaccination timepoints were stimulated for 18 hours with 5 HAU of either A/California or PR8, 2000 PFU of LCMV Armstrong, or were left unstimulated, then stained for IFNγ to detect influenza-responsive T cells. Other effector markers, TNFα and granzyme B, were also measured to assess function. (A) Representative strong (*top*) and weak (*bottom*) donor CD8 T cell responses against A/California. (B) Comparison of A/California and PR8 CD8 T cell responses on a per-donor basis as measured by IFNγ staining in select young (filled symbols) and 65+ (open symbols) donors. (C) Proportion of IFNγ positive CD8 T cells with multifunctional effector potential in A/California and PR8-responsive T cells.

CD8 T cells tend to be highly cross-reactive between strains and may limit disease severity in cases where neutralizing antibodies are absent [23], [54]. Ten pre-pandemic PBMC samples from the cohort, representing all age groups, were tested for T cell responses to the recent pandemic influenza, A/California/7/2009-like strain, to determine whether a functional cross-reactive memory CD8 T cell response could be elicited against a novel strain. Indeed, we found that regardless of age, for all donors tested, their CD8 T cell response to A/California stimulation measured as the frequency of IFNγ producing T cells was similar in magnitude as their response to A/PR8 stimulation (Fig. 6A,B). Phenotypes of functional ability were further examined in the CD8 T cell IFNγ^+^ subsets activated from the two strains (Fig. 6C). In both PR8 and A/California stimulations, about 1/3 of the influenza responsive CD8 T cells were also granzyme B^+^. Smaller proportions of IFNγ^+^ CD8 T cells were capable of co-producing TNFα, and the proportions of multifunctional T cells were similar after activation with either virus. Only a very small fraction of CD8 T cells were capable of producing all three effector molecules measured within 18h of stimulation. Therefore, although a significant proportion of T cells from older donors express markers that indicate an advanced state of differentiation sometimes associated with immune senescence [38], [55], there are still sufficient functional influenza-specific CD8 T cells to mount a cross-reactive immune response to the recent H1N1 strain. As suggested by others [56], such cross-reactive T cell populations may have contributed to the diminished disease severity observed during the 2009 influenza pandemic.

## Discussion

T cells in humans are well known to accumulate phenotypic and functional changes with age. For example, CD8 T cells in older humans are more likely to lack the costimulatory molecules CD28 and CD27 and express CD45RA as well as KRLG1 and CD57 [14], [30]–[34], [36]–[39], [57]. This phenotype has been attributed to the oligoclonal expansion of T cells that were stimulated repetitively over a lifetime to control persistently infecting pathogens such as CMV. Less clear however, is the effect of aging on the phenotype of T cells specific for an acutely infecting pathogen such as influenza virus. Here we have demonstrated that an acute infection can similarly result in the accumulation of late effector CD8 T cells that show markers that have been previously associated with immune senescence seen in late chronic infections such as with CMV.

The CD45RA marker is normally expressed on naïve T cells as well as late effectors. We detected CD45RA on IFNγ-producing influenza-specific T cells after only 18h of stimulation, a timepoint which is too short for the expansion of influenza-specific T cells from naïve precursors. Therefore, the CD45RA marker can be used to identify late effector cells. We found a higher proportion of the influenza responsive CD8 and CD4 T cells were of this late effector CD45RA^+^ phenotype, and that CD8 but not CD4 T cells had also significantly lost expression of costimulatory molecules CD28 and CD27. Similar findings have been made for CMV-specific T cells [34], [58], and the presence of CD45RA^+^ cells has also been noted in influenza-stimulated PBMC from older subjects [59]. The overall frequency of CD45RA^+^ influenza-specific CD8 T cells out of total CD8 T cells was similar in the old and the young; rather it was the proportion of CD45RA^+^ influenza-specific T cells out of the influenza-specific CD8 T cells that was higher, arguing that it was a loss of the less differentiated CD45RO cells that led to the higher proportion of CD45RA^+^ influenza-specific T cells in the older group.

By using A2/M1 tetramers to identify influenza specific T cells directly ex vivo we also observed that a higher proportion of influenza-specific CD8 T cells from older donors were double positive for KLRG1 and CD57, inhibitory receptors implicated in proliferative senescence [14], [16], [60]. CD27 and CD28 are also thought to be necessary for maintenance of telomerase function and the continued ability to proliferate upon stimulation [40], [61], [62]. T cell memory to influenza in the older group contains a significant proportion of cells expressing markers that have been attributed to immune senescence.

There are at least two possible explanations for why older donors' influenza-specific T cells would be enriched in more differentiated effector cells. They may have lost their RO memory T cells due to repetitive stimulation or these cells may be absent due to failure to receive sufficient survival signals for their maintenance over time, or a combination of the two. It has been previously observed that the influenza protein composition of the TIV varies depending on the manufacturer [63]. Since the Sanofi formulation of the seasonal TIV contains minimal internal viral protein compared to the levels of hemagglutinin and is optimized for its induction of antibody rather than T cell responses, older individuals' T cells may not have been sufficiently boosted in recent years. Indeed, as has been reported by others [64], [65], we saw little or no increase in the cross-reactive T cell response upon vaccination in the old or young. In Canada, seniors (65+) have been recommended to receive influenza vaccination since 1999 [66] and thus may have been repeatedly vaccinated and lost the opportunity for a natural infection to boost the T cell response. In a mouse model of viral infection, it was shown that secondary restimulation is required for high avidity antibodies and enhanced T cell functionality in old but not adult aged mice [67]. Whether the addition of T cell epitopes and appropriate adjuvants to the influenza vaccine administered earlier in life could improve the lifespan of functional influenza-specific T cells into old age remains to be determined.

A live attenuated influenza vaccine (LAIV) has been approved for people aged 5–49 in the United States and was recently also approved for use in Canada. Although live vaccines are expected to be more efficient in inducing cross-protective T cell responses, no protective benefit was shown for LAIV-vaccinated individuals aged 50 and over [68], [69]. It may be that LAIV may not provide additional protection to elderly individuals due to pre-existing IgA antibodies in the respiratory tract. It is also possible that the viral titers present in LAIV are not sufficiently high to robustly restimulate T cell memory. A recent study showed that in a mouse model, the inclusion of the CD8 T cell costimulatory ligand, 4-1BBL, in a single dose replication defective adenovirus vaccine containing influenza NP, resulted in sustained functional CD8 T cell responses in the lungs out to 6 months post-vaccination and prolonged protection against influenza induced disease [70]. Vaccines that incorporate both CD8 T cell epitopes and adjuvants that prolong CD8 T cell memory would therefore be of interest to improve influenza vaccines.

A second scenario that might explain the loss of functional memory T cells in older adults is that the healthy older donors may have encountered multiple influenza infections in their lifetime and this repeated reactivation, as in latent persistent infections, may have driven CD8 and CD4 T cells towards terminal differentiation and senescence. In support of this, Bucks et al. [71] showed that repetitive infection of mice with influenza virus can lead to functional exhaustion as measured by loss of IFNγ producing cells and upregulation of TNF receptor apoptosis-inducing ligand (TRAIL), without the upregulation of PD-1. This is similar to the phenotype we observe in influenza-specific T cells from older humans, which also resemble T cells specific for a persistent latent infection (CMV). In contrast, the cells in both the mouse influenza study and the present report are distinct from the PD-1^hi^ phenotype observed with viruses that maintain a chronically higher viral load, such as seen in HIV [43], [46]–[49] or HCV infection [50]. Consistent with these findings, a recent study demonstrated that PD-1 expression in healthy individuals does not correlate with the exhausted phenotype found during chronic infection, but rather with the effector memory population of CD8 T cells [72].

With the available data we cannot formally conclude whether repeat exposure to influenza virus and/or failure to boost memory T cells leads to this late differentiated phenotype. However, the ability to predict the response to influenza vaccination by examining only the influenza-specific population of KLRG1^+^CD57^+^ CD8 T cells but not the total CD8 T cell pool leads us to suggest that these observations may reflect a specific effect of multiple rounds of exposure to influenza.

Despite the late differentiation state in healthy older adults, cross-reactive T cell populations capable of responding to a new influenza virus, A/California/7/2009, persisted. As many individuals in the population may not possess sufficiently protective levels of neutralizing antibody against A/California, cross-reactive CD8 T cells could have ameliorated disease severity in these individuals. Evidence of cross-reactivity between influenza-responsive memory CD8 T cells was found through in vitro challenge with A/California, a virus that, at the time of sample collection (in the fall of 2008), we presume none of the donors had been exposed. There have also been multiple studies which have identified pre-existing cross-reactive neutralizing antibodies to A/California in older populations, which when combined with CD8 T cell responses, may have been responsible for the lack of infection seen in the older population during the 2009 pandemic [73]. However, similar to seasonal influenza, those older people who were infected with influenza tended to show severe outcomes [74]. As has been suggested by others [56], these cases of older individuals who did acquire illness from A/California may have been those with the most functionally senescent T cell memory to influenza virus.

Accumulations of dysfunctional KLRG1^+^ and KLRG1^+^CD57^+^ CD8 T cells specific for EBV [75] and CMV [14], [16], [76] have been previously noted in aging and indeed the presence of oligoclonal expansions of CMV-specific T cells have been cited as a way of determining the functional immune age of the immune system [4], [75]. However, it is difficult to assess whether the CMV infection actually contributes to the aging of the immune system or simply that as the immune system ages and T cells become less effective at controlling chronic infections, viral reactivation becomes more frequent, leading to further clonal expansion of the CMV-reactive T cells and their terminal differentiation. In contrast, as influenza is an acute infection without the issue of reactivation within the individual, the state of the influenza-specific memory T cell population may represent a more useful prognostic indicator of a healthy immune system independent of the effects of chronic infection and inflammation on aging and mortality, with the caveat that the state of these cells may also reflect the history of influenza exposure.

There is currently much interest in understanding the correlates of a healthy immune system. As pointed out by others [10], [77], to date we have only minimal information about what features of the immune system should be measured in order to evaluate this point. In this study, the vaccination of a healthy older cohort resulted in a range of antibody responses, thereby offering the opportunity to compare pre-vaccination influenza-specific T cell frequency and phenotype with the response to the vaccine. In the younger group, the frequency of HLA-A2/M1 tetramer positive T cells pre-vaccination correlated well with the subsequent H1-specific antibody response to the seasonal H1N1 contained in the vaccine. Paradoxically, the older group actually showed a decreased response the higher the pre-vaccination tetramer^+^ T cell frequency. However, further analysis indicated that this negative correlation was largely due to the presence of a high proportion of “senescent” phenotype KLRG1^+^CD57^+^ cells that were mostly absent in younger donors. CD8 T cell responses are generally thought not to contribute to antibody responses and yet the data show a correlation between the state of the cross-reactive CD8 T cell response (to internal viral proteins) pre-vaccination and the subsequent antibody response to the 2007 H1. Thus by comparing the influenza-specific CD8 T cell phenotype pre-vaccination with the antibody response to the vaccine, we may instead be getting a read-out of the general robustness of the immune system. Indeed, others have shown that expansions of the total CD8^+^CD28^−^ T cell population in older individuals correlates with a poor vaccination outcome [78], [79]. On the other hand, we cannot rule out that these findings reflect the specific exposure of individuals to multiple influenza viruses over their lifespan, leading to a desensitization of both antibody and T cell responses to influenza.

Several recent studies, using either granzyme or cytokine release from total PBMC upon restimulation with influenza virus, have suggested that older adults have less functional responses to influenza virus than younger adults and that cell-mediated immunity is a better predictor of vaccine responsiveness or protection from influenza virus than are antibody responses [25], [26], [59]. However, we find that if anything the older donors have generally higher levels of granzyme B than the younger donors, consistent with their accumulation of more late effector cells. Although the previous studies did not specifically gate on influenza-specific T cells and therefore may also have included NK cell responses, the present study agrees with previous findings suggesting that cell-mediated immunity is a better predictor of vaccine responses than pre-existing antibodies. We found that influenza-specific IFNγ production by CD4 and perhaps CD8 T cells or the absence of the KLRG1^+^CD57^+^ influenza-specific tetramer positive population each independently predict influenza antibody responses upon vaccination. Vaccine responsiveness may be a reflection of a healthy older immune system [25], [59].

In sum, we have shown that in older persons the memory T cells specific for an acutely infecting virus, influenza, exhibit a loss of the less differentiated memory population and enrichment of memory T cells exhibiting a late differentiation phenotype that has been previously associated with immune senescence. It remains to be determined whether these late effectors are indeed senescent or could be rescued by appropriate boosting. We further show that the influenza vaccine responsiveness of older adults is predicted by the status of their influenza-specific memory CD8 T cells.

## Supporting Information

Figure S1 **PD-1 expression is elevated in both influenza-specific and total CD8 T cells from older individuals**. PD-1 levels were measured by flow cytometry direct ex vivo in conjunction with tetramer staining for HLA–A2 positive young and older donors. *Left:* Median fluorescence intensity (MFI) of PD-1 in influenza M1-specific CD8 T cells. *Right:* Representative PD-1 expression in tetramer^+^ and total CD8 T cells compared to FMO control (shaded) in under 40 and 65+ donors.
(TIF)
