# Supplementary figures and images for: Influenza-Specific T Cells from Older People Are Enriched in the Late Effector Subset and Their Presence Inversely Correlates with Vaccine Response
Source: PLoS One. 2011 Aug 22;6(8):e23698. doi: 10.1371/journal.pone.0023698 (PMC3161762; doi:10.1371/journal.pone.0023698)

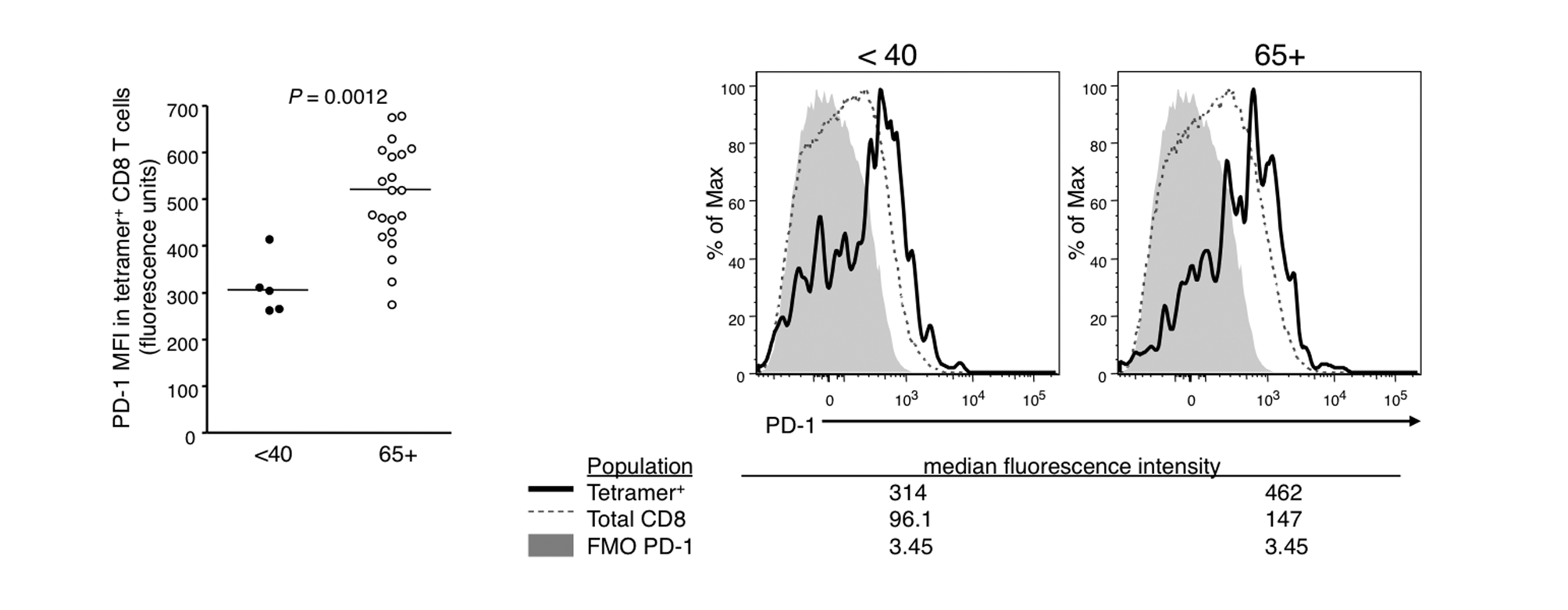

Supplement: Figure S1 — PD-1 expression is elevated in both influenza-specific and total CD8 T cells from older individuals. PD-1 levels were measured by flow cytometry direct ex vivo in conjunction with tetramer staining for HLA–A2 positive young and older donors. Left: Median fluorescence intensity (MFI) of PD-1 in influenza M1-specific CD8 T cells. Right: Representative PD-1 expression in tetramer+ and total CD8 T cells compared to FMO control (shaded) in under 40 and 65+ donors. (TIF) [file pone.0023698.s001.tif]
